# Age-related immunosenescence and Epstein–Barr virus-positive mucocutaneous ulcer presenting as oral cancer: a case report

**DOI:** 10.3389/fmed.2024.1458606

**Published:** 2025-01-22

**Authors:** Jiayuan Ye, Shengqiang Ji, Zhouxiao Wu, Genhua Ma, Jing Chen, Shimin Wu, Yilian Xie

**Affiliations:** ^1^Department of Infectious Diseases, Shangyu People’s Hospital of Shaoxing, Shaoxing University, Shaoxing, China; ^2^Ningbo Clinical Pathology Diagnosis Center, Ningbo, China; ^3^Health Science Center, Ningbo University, Ningbo, China; ^4^Department of Dermatology, Shangyu People’s Hospital of Shaoxing, Shaoxing University, Shaoxing, China; ^5^Department of Gastroenterology, Shangyu People’s Hospital of Shaoxing, Shaoxing University, Shaoxing, China; ^6^Department of Infectious Diseases, The First Affiliated Hospital of Ningbo University, Ningbo, China

**Keywords:** Epstein–Barr virus, Epstein–Barr virus-positive mucocutaneous ulcer, age-related immunosuppression, mandibular necrosis, case report

## Abstract

Epstein–Barr virus-positive mucocutaneous ulcer (EBVMCU) is a rare condition characterized by skin or mucosal lesions resulting from defective immune surveillance of EBV due to immunosuppression, which can be iatrogenic or age-related. It represents a benign lymphoproliferative disorder that clinically may mimic malignant tumors. However, EBVMCU typically progresses slowly and often resolves spontaneously without specific treatment, emphasizing the critical need for differential diagnosis from malignancies. In this study, we report a case of EBVMCU in an elderly patient demonstrating ulcers on the oral mucosa, buccal area, and maxillary mucosa, with associated bone destruction, which was initially suspected to be an oral malignancy but was confirmed as EBVMCU through biopsy. This case underscores the importance of considering EBVMCU in elderly patients with unexplained persistent mucosal ulcers to exclude malignancies. In addition, attention should be given to unhealthy lifestyle habits such as smoking and drinking, as well as poor oral hygiene, which are potential factors that increase the risk of this disease and contribute to worse prognoses.

## 1 Introduction

EBVMCU was described as a rare disease in 2010 (1). In the 5th edition (2022) of the World Health Organization (WHO) classification of hematolymphoid tumors, EBVMCU is classified under lymphoproliferative disorders and lymphomas associated with immunodeficiency and immune dysregulation (2). EBVMCU is considered an indolent tumor with self-limiting clinical features related to EBV infection. It often occurs in the context of drug-induced immunosuppression, age-related immunosenescence, and primary or acquired immunodeficiency, where EBV-infected cells can evade immune surveillance and proliferate, leading to EBV-related lymphoproliferative disorders (3).

EBV, also known as human herpesvirus 4, is a common virus primarily transmitted through saliva (4). It persistently infects B cells in over 95% of adults, resulting in asymptomatic lifelong carriage (5). When EBV disrupts host immune homeostasis, it can lead to a spectrum of Epstein–Barr virus-associated B-cell lymphoproliferative disorders (EBV-B-LPDs), ranging from indolent to lymphomas. These include Burkitt lymphoma (6), Hodgkin lymphoma (7), post-transplant lymphoproliferative disorder (8), and Epstein–Barr virus-positive mucocutaneous ulcer (EBVMCU), among others.

EBVMCU was first characterized as an Epstein–Barr virus-associated B-cell lymphoproliferative disorder in 2010 by Dojcinov et al., who reported a series of cases involving mucosal sites of the oropharynx, skin, and gastrointestinal tract (1). EBVMCU primarily arises due to immunosuppression, which can be age-related immunosenescence and iatrogenic immunosuppression induced by therapies such as methotrexate, cyclosporine, and azathioprine, as well as steroid treatments (9). While it is rarely observed in individuals with primary immunodeficiencies, it is more frequently noted in solid organ or bone marrow transplant recipients, HIV-positive patients, and individuals undergoing treatment for other lymphomas or tumors (10, 11).

EBVMCU presents as well-defined ulcers in the oral cavity, gastrointestinal tract, or skin, characterized by shallow, solitary, and painful ulcers with distinct borders. There are no associated systemic symptoms or involvement of other sites (12). Due to the non-specific nature of ulcerative lesions, diagnosing EBVMCU poses a challenge, making it difficult to distinguish it from other neoplastic conditions. Research indicates that the median age of EBVMCU patients is 71 years, with 27% of reported cases occurring in patients aged 60 years or older, without known immunodeficiency (9). Immunohistochemical studies of ulcer tissues have shown positivity for CD20, CD30, EBER, MUM-1, OCT-2, and PAX-5 in monoclonal B-immunoblasts (13). Therefore, persistent skin ulcers in elderly patients with age-related immunosenescence should raise suspicion for EBVMCU.

In this study, we report a case of EBVMCU in an elderly patient with age-related immunosenescence to improve clinical recognition and reduce the risk of misdiagnosis and missed diagnosis.

## 2 Case report

**Figure 1:**
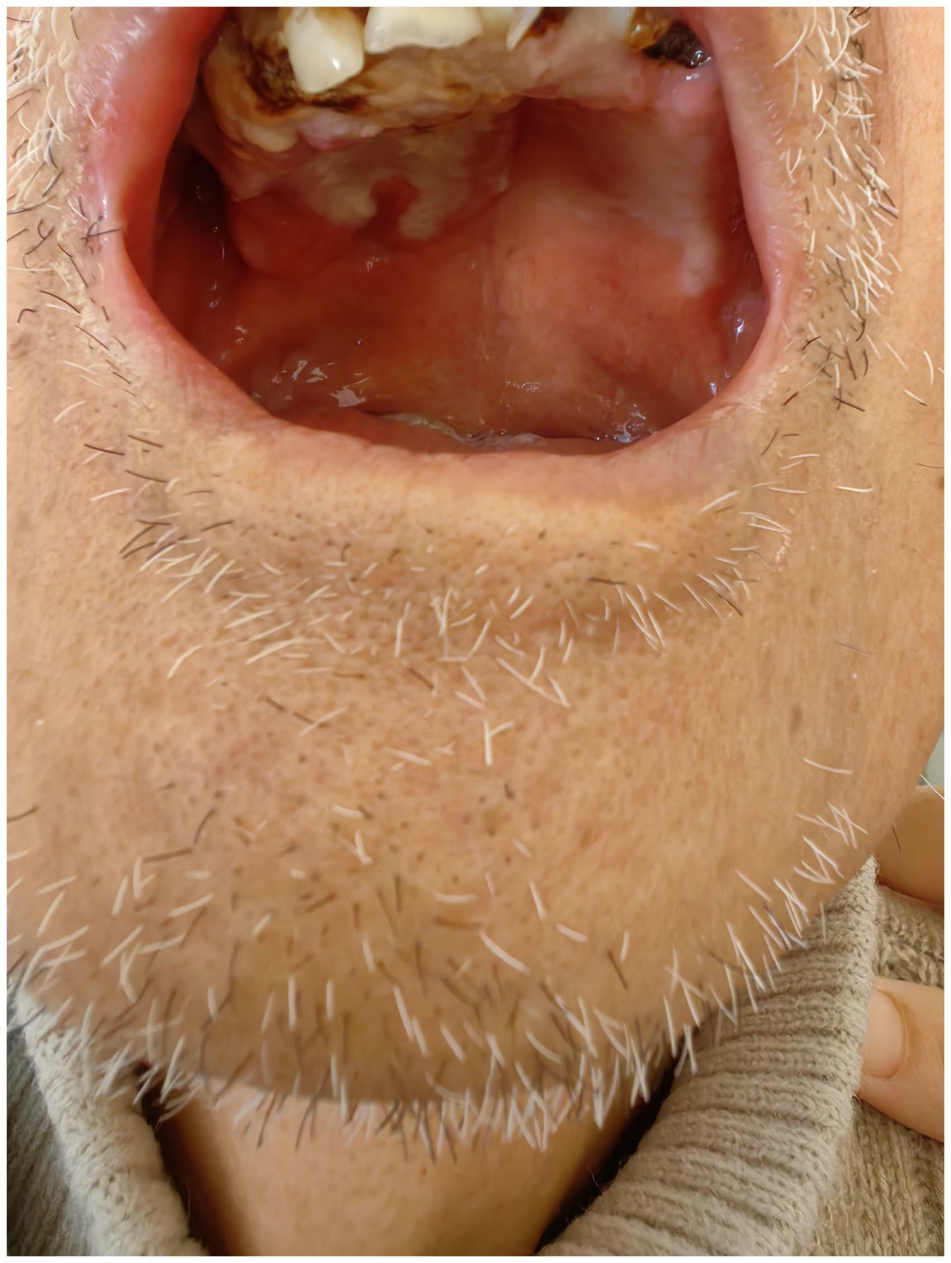
During the initial visit, the patient presented with extensive purulent deposits on the tooth surfaces, accompanied by multiple residual roots. In addition, significant ulceration was noted on the mucosa of the cheeks and upper palate.

The patient is an 84-year-old man from China who presented with persistent ulcerations of the gums, buccal mucosa, and maxillary mucosa for 3 months. He was admitted to the Department of Infectious Diseases at the First Affiliated Hospital of Ningbo University. Three months ago, the patient developed ulcers on the maxillary gums and mucosa, accompanied by local purulent plaques (Figure 1). Sequential treatments with intravenous cefmetazole (2.0 g q12h) and oral cefdinir capsules (0.1 g q8h) at previous hospitals showed no improvement, prompting admission to our hospital. The patient had a history of hypertension for over 10 years, well-controlled with oral indapamide 1.5 mg daily. He had a smoking history of over 20 years, averaging 10 cigarettes per day, and an alcohol consumption history of over 30 years, averaging 50 g of liquor daily.

On admission examination, the patient presents with normal facial symmetry and a mouth opening to three fingers’ width with a normal opening type. However, the patient has extremely poor oral hygiene, characterized by numerous purulent plaques, soft calculus deposits on teeth surfaces, and multiple residual roots in the oral cavity. Additionally, there is a yellow pseudomembrane covering various surfaces, along with extensive irregularly shaped erosions and ulcers with unclear borders and tenderness. The upper palate mucosa shows concave surfaces.

**Figure 2:**
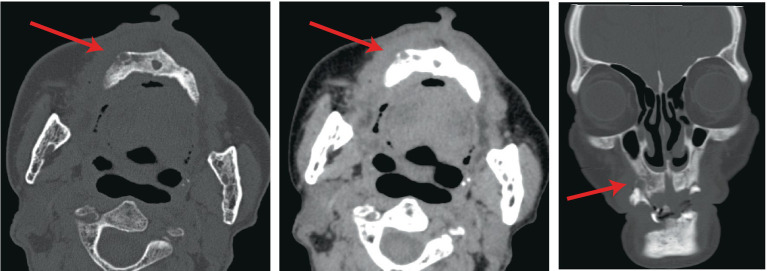
A plain computed tomography scan of the maxillofacial region indicated local bone destruction with surrounding soft tissue swelling on the right upper palate.

**Figure 3:**
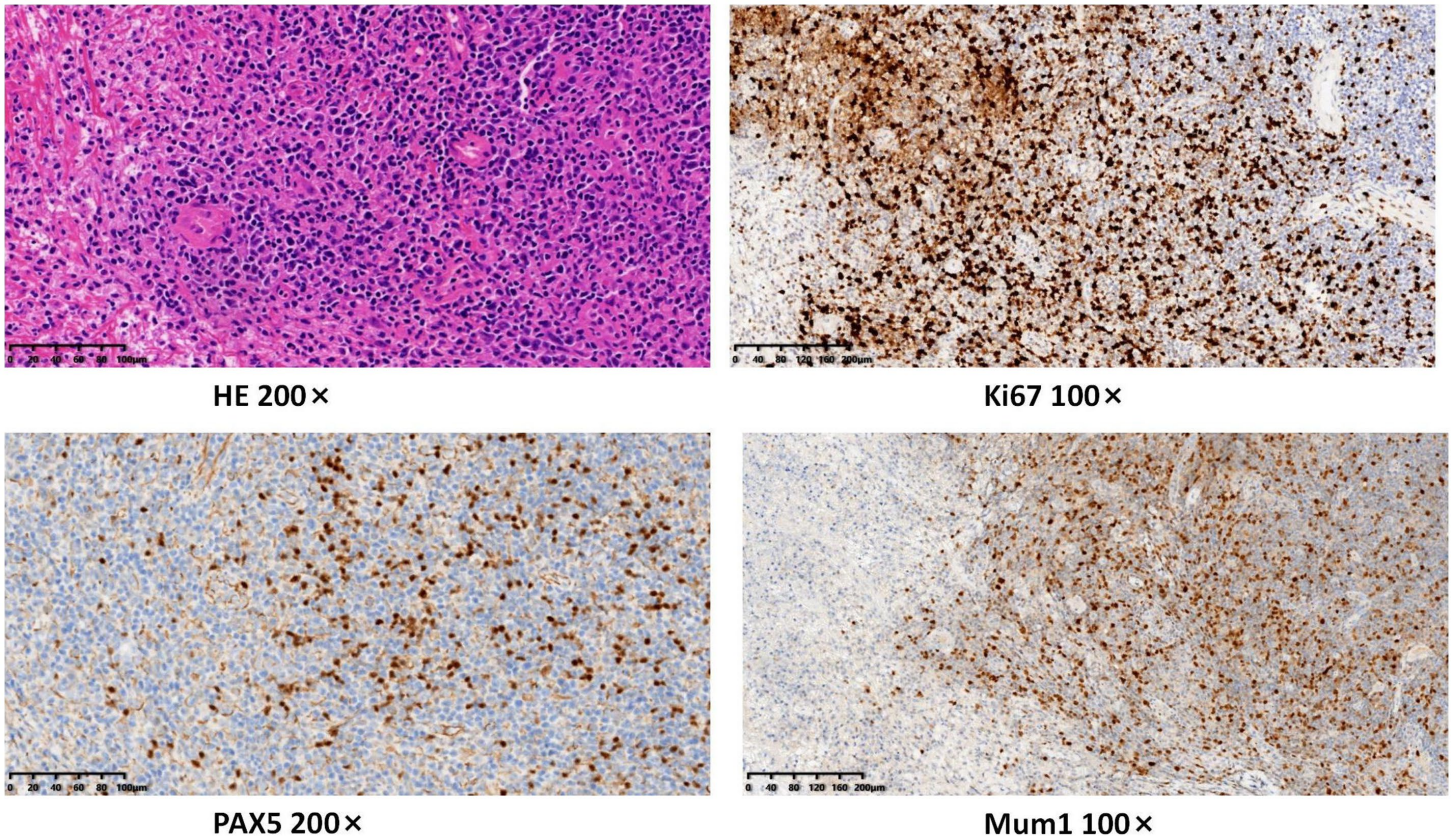
The tissue biopsy results revealed diffuse proliferation of atypical lymphocytes of varying sizes. Immunohistochemical results indicated PAX-5 (cells+), Mum-1 (plasma cells+), and Ki-67 (ulcer area+, 20%).

**Figure 4:**
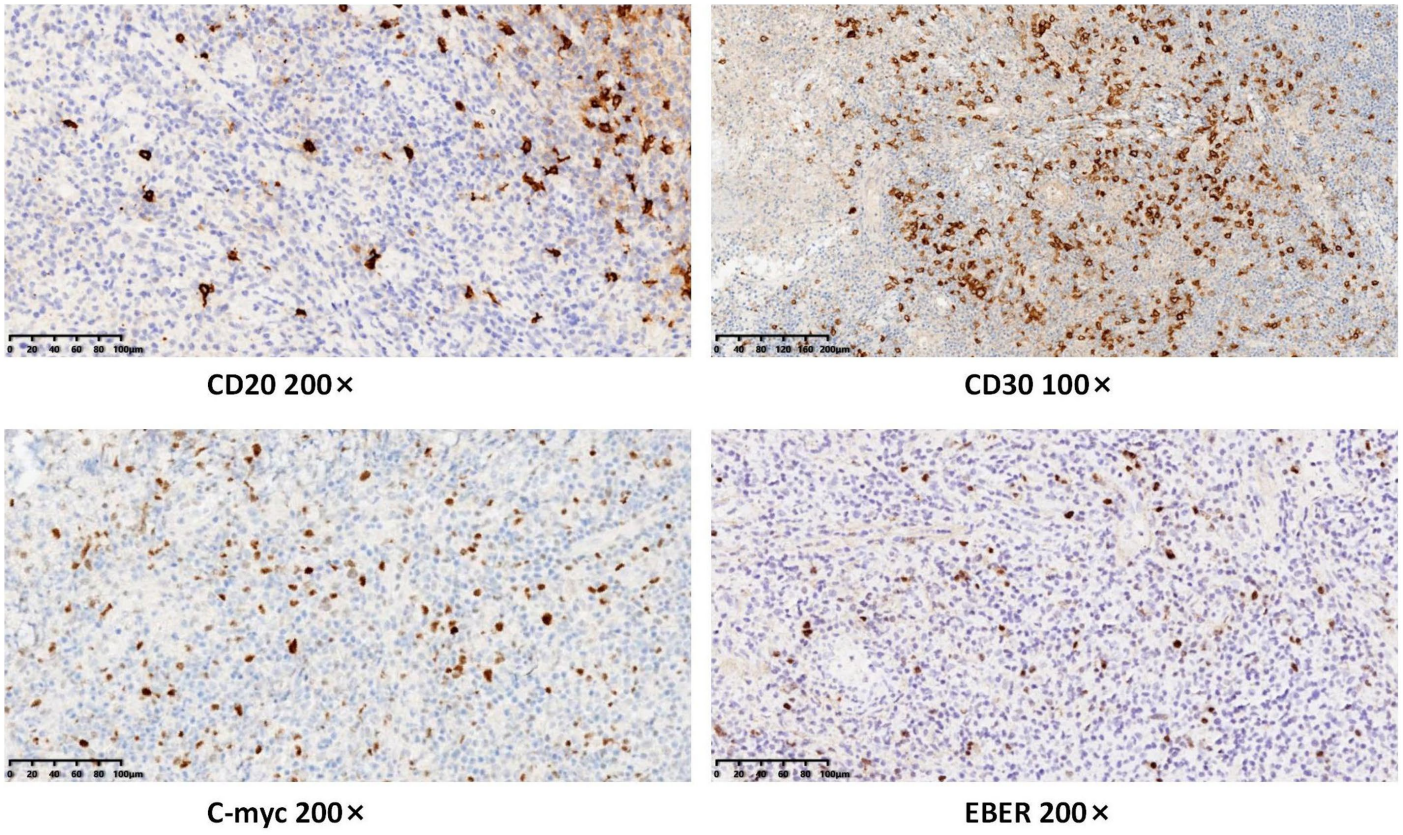
Immunohistochemical results indicated CD20 (B cells+), CD30 (+), C-myc (<5%), and EBER (+).

Following admission, comprehensive investigations were performed, and detailed laboratory results are shown in Table 1. A plain computed tomography scan of the maxillofacial region indicated local bone destruction with surrounding soft tissue swelling on the right upper palate, prompting clinical correlation and biopsy to exclude neoplasms (Figure 2). The patient was treated with meropenem (1 g q8h) for anti-infective therapy and received oral hygiene measures, with inadequate response. A subsequent positron emission tomography/computed tomography scan revealed bone destruction of the maxilla with surrounding soft tissue shadows, as well as soft tissue shadows in the left nasal cavity. Increased FDG metabolism was noted in these lesions, suggesting the presence of malignant tumors. Lymphoma should be ruled out, and a soft tissue biopsy was recommended for further evaluation. Following a tissue biopsy, the mucosal ulcers and significant lymphoid tissue hyperplasia were evaluated. Immunohistochemical results (Figures 3, 4) indicated CD79a (B cells+), CD20 (B cells+), Mum-1 (plasma cells+), Ki-67 (ulcer area+, 20%), EBER (+), PAX-5 (cells+), C-myc (<5%), and CD30 (+), suggesting EBV-positive mucocutaneous ulcers. Subsequently, we discontinued antibiotic therapy and advised the patient to maintain oral hygiene, cease smoking and alcohol consumption, and undergo follow-up observation. After a 3-month follow-up, it was found that the patient’s disease continued to progress. The persistent oral ulcer caused difficulty in eating, which led the patient to discontinue treatment, and eventually the patient passed away.

**Table 1:** The physical examination data and laboratory test results.

Tests	Result	Reference range	Interpretation
Patient’s vitals			
BP(mmHg)	116/86	<120/80	Normal
HR(beats/min)	85	60–100	Normal
RR(breaths/min)	16	Dec-20	Normal
T(°C)	36.5	37	Normal
CBC test			
WBC(10*9/L)	4.9	3.5–9.5	Normal
NEU(10*9/L)	2.59	1.8–6.3	Normal
LYM(10*9/L)	1.6	1.1–3.2	Normal
RBC(10*9/L)	4	4.3–5.8	Normal
HGB(g/l)	124	130–175	Low
MCV(fl)	90.5	82–100	Normal
PLT(10*9/L)	198	125–350	Normal
CRP(mg/l)	11.8	<8	High
ESR(mm/h)	50	0–15	High
Laboratory chemistry			
ALT(U/L)	17.8	Sep-50	Normal
AST(U/L)	35.4	15–40	Normal
TBIL (μmol/L)	17	4.5–22	Normal
DBIL (μmol/L)	5.3	0–6	Normal
IBIL (μmol/L)	12.7	1.5–14	Normal
PCT(ng/mL)	0.07	<0.046	High
Ferritin(ng/mL)	410.7	30–400	High
Immune indicators			
1,3-beta-D-glucan(pg./ml)	<37.5	<37.5	Negative
Galactomannan	0.07	<0.5	Negative
EBV-NA-IgG(U/mL)	78.6	>20	Positive
EBV-VCA-IgG(U/mL)	>750	0–20	Positive
EBV-EA-IgM(COI)	1.59	<0.9	Positive
EBV-DNA(copies/mL)	BDL	400	Negative
CMV-DNA(copies/mL)	BDL	400	Negative
CMV IgM(U/mL)	4.5	<20	Negative
ANA	Negative	<1:100	Negative

BP, blood pressure; HR, heart rate; RR, respiration rate; T, temperature; WBC, white blood cells; NEU, neutrophil; EOS, eosinophil; LYM, lymphocyte; RBC, red blood cells; HGB, hemoglobin; MCV, mean corpuscular volume; PLT, platelet; ESR, erythrocyte sedimentation rate; ALT, alanine aminotransferase; AST, aspartate aminotransferase; TBIL, total bilirubin; DBIL, direct bilirubin; IBIL, indirect bilirubin; PCT, procalcitonin; ANA, antinuclear antibodies.

## 3 Discussion

Our patient presented with persistent gingival and palatal mucosal ulceration lasting 3 months. After conventional anti-infective treatment proved ineffective, a maxillofacial CT scan revealed localized bone destruction. The primary differential diagnosis focused on excluding oral cancer. Oral cancer often manifests as persistent ulcers lasting more than 3 weeks, typically accompanied by indurated edges, erythroleukoplakia (red and white patches), nodules, and bone destruction. These features are frequently associated with risk factors such as smoking, alcohol consumption, and poor oral hygiene (14, 15). Unlike the typical characteristics of EBVMCU, our patient’s clinical presentation was more suggestive of oral cancer, especially given the presence of high-risk factors. However, pathological examination ultimately indicated lymphoid hyperplasia, and immunohistochemistry confirmed features consistent with EBVMCU. As a result, we excluded the diagnosis of oral cancer.

In over 90% of EBVMCU cases, there is an underlying primary or secondary immunodeficiency (1). The literature suggests that unhealthy habits, such as long-term smoking and alcohol consumption, may weaken the immune system, impairing the body’s ability to control EBV and potentially increasing the risk of EBV-related diseases (16). The primary cause of age-related EBVMCU is thought to be the gradual decline in immune system function with aging. This decline includes thymic atrophy, reduced production of naïve T cells, accumulation of activated memory T cells, decreased lymphocyte cytokine secretion, impaired antigen-presenting cell function, and chronic inflammation. These changes lead to diminished T-cell activity in response to antigen stimulation (16, 17). A direct consequence of this immunosenescence is the potential reactivation of EBV, which can result in EBVMCU (3). EBVMCU lesions can occur at various body sites but are most commonly found in the oropharyngeal mucosa (10, 18). This distribution is attributed to the high concentration of EBV-infected B cells in Waldeyer’s ring and the oral cavity’s propensity for microtrauma to mucosal surfaces due to food debris and dentures (19). Our patient, an 84-year-old man, had no history of immunosuppressant use or immunodeficiency-related diseases, making this a clear case of age-related EBVMCU. In addition, the patient exhibited extremely poor oral hygiene and a long history of smoking and alcohol consumption. Coupled with age-related immune decline, these factors likely created a susceptible environment for the development of EBVMCU.

EBVMCU is typically described as an indolent, self-limiting disease with a benign clinical course, often requiring no treatment (20). For age-related EBVMCU, current treatment and prognosis recommendations prioritize follow-up monitoring. It is advised to observe for 2–8 weeks, with chemotherapy, radiotherapy, or surgical intervention recommended only if the lesions remain unchanged or progress during follow-up (11). Regarding prognosis, studies report that 80% of EBVMCU cases achieve remission, with only 1% resulting in mortality due to disease progression (9). In a follow-up study of 34 EBVMCU patients, all demonstrated favorable outcomes after either immunosuppressant reduction or chemotherapy (21). In addition, a systematic review of 186 EBVMCU cases found that during a follow-up period (ranging from 1 to 180 months), 8 cases showed spontaneous ulcer resolution, and 126 achieved complete or partial remission after stopping or reducing immunosuppressants, chemotherapy, or radiotherapy (10). However, other studies suggest different approaches. Roberts et al. argue that although many EBVMCU cases exhibit a self-limiting course, the disease may also present as a debilitating, persistent condition requiring active treatment to prevent progression (22). In our case, the patient initially opted for close monitoring. Unfortunately, due to the progressive nature of the oral ulcerations, the patient missed the optimal treatment window and ultimately succumbed to the condition after forgoing further treatment. Currently, no evidence-based guidelines or expert consensus exists for EBVMCU treatment. However, based on the unfavorable outcome of this case, we suggest that early intervention may be warranted for age-related EBVMCU. A comprehensive assessment of the patient’s overall health, immune status, and lifestyle factors may help prevent disease progression and avoid missed opportunities for effective treatment.

In conclusion, we present a unique case of EBVMCU involving the buccal mucosa and upper palate with bone destruction, initially misdiagnosed as oral malignancy but confirmed through biopsy. Unlike the majority of EBVMCU cases, which exhibit spontaneous remission, our patient experienced progressive ulceration and eventually succumbed to the disease. This case highlights the potential impact of lifestyle factors, such as oral hygiene, smoking, and alcohol consumption, on disease progression. It also raises questions about the optimal management strategy for age-related EBVMCU, as current evidence suggests that the majority of cases are resolved with close monitoring. Further large-scale studies are needed to clarify treatment protocols for age-related EBVMCU and assess the influence of lifestyle factors on prognosis. Close collaboration between clinicians and pathologists is essential to prevent misdiagnosis and ensure timely intervention.

## Data Availability

The original contributions presented in the study are included in the article/supplementary material, further inquiries can be directed to the corresponding authors.
